# Global collaborations in antimicrobial stewardship: All hands on deck

**DOI:** 10.1017/ash.2023.122

**Published:** 2023-04-05

**Authors:** Diane Ashiru-Oredope, Bradley J. Langford, Candice Bonaconsa, Vrinda Nampoothiri, Esmita Charani, Debra A. Goff

**Affiliations:** 1 HCAI, Fungal, AMR, AMU & Sepsis Division, UK Health Security Agency, London, UK; 2 School of Pharmacy University of Nottingham, Nottingham, UK; 3 Health Protection, Public Health Ontario, Toronto, Ontario, Canada; 4 Dalla Lana School of Public Health, University of Toronto, Ontario, Canada; 5 Division of Infectious Diseases and HIV Medicine, Department of Medicine, Groote Schuur Hospital, University of Cape Town, Cape Town, South Africa; 6 Department of Health Sciences Research, Amrita Institute of Medical Sciences, Amrita Vishwa Vidyapeetham University Kochi, Kerala, India; 7 Department of Infection Control and Epidemiology, Amrita Institute of Medical Sciences, Amrita Vishwa Vidyapeetham University, Kochi, Kerala, India; 8 Faculty of Health and Life Sciences, University of Liverpool, Liverpool, UK; 9 The Ohio State University Wexner Medical Center, The Ohio State University College of Pharmacy, Columbus, Ohio, USA

## Abstract

Tackling antimicrobial resistance (AMR) through antimicrobial stewardship (AMS) interventions is a key objective within the World Health Organization (WHO)’s Global Action on AMR. We outline the reasons why global collaborations for AMS are needed. We provide examples of global collaborations, and we offer considerations when starting on a global health journey focused on AMS.

Antimicrobial resistance (AMR) has significant impacts across geographic borders for human health as well as veterinary medicine, agriculture, and the environment. It is therefore an important One Health issue. Conserving the effectiveness of antimicrobial agents and optimizing their use through antimicrobial stewardship (AMS) is 1 of 5 objectives of the WHO Global Action Plan on AMR.

Although the term global health is often associated with work linked to low- to middle-income countries (LMICs), any collaborative transnational research and actions promoting health for all constitute global health.^1^ Multiple-country collaborations and bidirectional learning across different income settings are essential for building and strengthening capacity for AMS interventions. Although variations in the composition of AMS teams, their functions, activities, and challenges exist, opportunities can be gleaned from the lessons of existing partnerships in AMS.

In this article, we highlight some examples of international collaborative AMS efforts that the authors have been involved in, and we identify opportunities for stewards to learn from and apply in their own practices.

## International collaborative AMS efforts

### Joint Programming Initiative on Antimicrobial Resistance (JPIAMR)

The priority areas of focus for JPIAMR, a collaborative platform engaging the European Commission and 29 member countries representing 5 continents, include AMS efforts such as improving diagnostics, therapeutic regimens, and surveillance (Table 1). Examples of impactful JPIAMR efforts so far include the development of a framework for optimal design of antimicrobial stewardship intervention research and prioritization of research areas for AMS behavioral initiatives in hospitals.

**Table 1.** Additional Examples of Global Collaborations Focused on Antimicrobial Stewardship

Countries Involved	Name of Project and Webpage plus Brief Information on Collaboration	Setting	Specific AMS TOPICS Collaborated on What Was Done (eg, surveillance, education & training)	Names of Lead Organizations	Current Status and Sustainability Plans	Healthcare Professionals Involved	Further Details
United Kingdom Nigeria Tanzania Zambia Kenya Malawi Sierra Leone Ghana	Commonwealth Partnerships for Antimicrobial Stewardship https://commonwealthpharmacy.org/publications/ https://commonwealthpharmacy.org/cwpams/	Hospitals mainly but also some community pharmacies	Antimicrobial use surveillance (global point-prevalence survey) Education and training prescribing app	Several hospitals in the United Kingdom and the African countries Program funded by UK Aid Fleming Fund since 2018 and managed by the Commonwealth Pharmacists Association (CPA) and the Tropical Health and Education Trust (THET)	Ongoing. Sustainability plans have included working closely with national pharmacy bodies in each of the countries. All projects are required to include their sustainability plans.	Pharmacists, doctors, nurses	26 health partnerships (12 in CwPAMS 1 and 14 in CwPAMS Extension) involving UK institutions and institutions in 8 African countries (Ghana, 6; Uganda, 6; Tanzania, 1; Zambia, 2; Kenya, 1; Malawi, 1; Sierra Leone, 1; Nigeria, 1). 6,544 (3,112 CwPAMS 1 and 3,432 CwPAMS extension) healthcare workers trained in AMS, IPC • >67 guidelines and protocols rolled out across the 8 countries • 20 peer-reviewed publications • >36 point-prevalence survey sites • >200 UK and in-country team members were involved in working through the partnerships to build capacity • 29 Global Health Pharmacy Fellows underwent a year-long leadership training year Evaluation of the first phase of the program highlighted that it was highly efficient and very effective and using the Organisation for Economic Cooperation and Development Assistance Committee (OECD-DAC) evaluation criteria was 82% (very good).
Nigeria Tanzania Zambia Kenya Malawi Sierra Leone Ghana United Kingdom	Development and implementation of Global AMS Game https://www.amsgame.com/ https://www.mdpi.com/2079-6382/11/5/611	Multiple settings	Education and training AMS Game	Commonwealth Pharmacy Association, Focus Games and THET	Completed. The project is sustained through ongoing promotion by CPA and THET and an online platform.	Pharmacists, doctors, nurses, project managers, non HCWs	The game was cocreated with a diverse group of stakeholders including national and frontline health professionals from high- and low-income countries. The feedback from the initial players (health professionals) of the game highlighted that the game is enjoyable. Also, that it provides an innovative and engaging opportunity for the players to discuss topics in AMR and AMS while improving and strengthening their knowledge of key topics.
29 countries from five continents	Joint Programming Initiative on Antimicrobial Resistance (JPIAMR) https://www.jpiamr.eu/	Multiple settings	Audit and feedback in primary care, antimicrobial stewardship in farm animals, consensus group on design analysis and reporting of antibiotic stewardship trials	European Commission and 29 member countries	Ongoing. Supported by the European Commission. Sustainability plan includes financial support and a governance structure.	Multi-disciplinary	International collaborative platform aiming to coordinates international research funding and collaborative action and collaborative AMR research
United Kingdom, India, South Africa	Antibiotic use across Surgical Pathways – Investigating, Redesigning and Evaluating Systems (ASPIRES) https://www.imperial.ac.uk/arc/aspires/	Hospitals	Capacity strengthening, ethnographic research, surveillance (as part of ASPIRES interventions)	Imperial College London, University of Cape Town, Amrita Institute of Medical Sciences	Completed. This research collaboration led to PhD studentships and fellowship applications for early-career researchers and opportunities for research funding.	Pharmacists, doctors, nurses, microbiologists, anthropologists, non-HCWs (ie, patients, caregivers)	Ethnographic study undertaken revealed various antibiotic prescribing behaviors and communication patterns within surgical team.
India, United States	Clinical efficacy and pharmacokinetics of colistimethate sodium and colistin in critically ill patients in an Indian hospital with high endemic rates of multidrug-resistant gram-negative bacterial infections: a prospective observational study https://www.ijidonline.com/article/S1201-9712(20)30638-X/fulltext	Hospitals	Study on pharmacokinetics of colistimethate sodium and colistin	Amrita Institute of Medical Sciences, University of Michigan	Completed. Provided a platform for further research and partnerships across participating sites.	Doctors, virologists, pharmacists, data analyst	Development of local dosing guidelines for colistin
Latin America, United States	Creating a Latin America network for clinical training and support of pharmacists involved in ASPs	Hospitals in 6 countries	Education and training	Universidad El Bosque, The Ohio State University	Ongoing. Sustainability plans include all projects required local doctors and pharmacists to work collaboratively during the projects and after completion.	Doctors, pharmacists, nurses	Developed new relationships between AMS team members. Each member contributed to the antibiotic hangtime project.
Lebanon, United States	A collaboration in a national neonatal antimicrobial stewardship program among 6 NICUs	Hospitals	Develop, implement, and sustain a national NICU ASP through a collaborative Lebanon–USA train-the-trainer ASP model to decrease antibiotic use	American University of Beirut Medical Center, Nationwide Children’s Hospital, The Ohio State University	Completed. NICU-ASP interventions are sustained by ongoing efforts by local thought leaders.	Doctors, pharmacists, microbiologists	Developed local guidance for antibiotics and cultures
South Africa, United States	A prospective study to improve duration of antimicrobial therapy in culture negative sepsis in NICUs in public and private hospitals across South Africa	Public and private hospitals in South Africa	Provided baseline NICU-ASP training to study participants who conducted audit and feedback interventions	The Ohio State University, Nationwide Children’s Hospital, and 16 South African hospitals	Completed. The study has led to sustained NICU ASP collaboration in South Africa	Pharmacists, doctors, nurses, microbiologists, infection preventionists	This is the first multidisciplinary national NICU-ASP study with interventions made by noninfectious diseases pharmacists within their existing positions.

### The Ohio State University (OSU) and Nationwide Children’s Hospital Partnerships for Global Antimicrobial Stewardship

A partnership between OSU and South African Society of Antimicrobial Stewardship (SAASP) implemented a Train the Trainer (TTT) Antimicrobial Stewardship Program for South African pharmacists.^2^ The TTT program included bilateral site visits, development of pharmacists’ AMS skills as well as research and publication. Mentees transferred their AMS skills to train an additional 120 pharmacists with no prior AMS experience. These pharmacists made >40,000 AMS interventions in 60 hospitals and coauthored AMS publications.

Another collaboration formed between the Centers for Disease Control and Prevention, OSU, Nationwide Children’s Hospital (NCH), and SAASP to implement neonatal antibiotic stewardship TTT pharmacist program.^2^ A NICU ASP tool kit was developed for ongoing training of additional pharmacists. This effort led to a research collaboration with physician members of the South Africa National Neonatal Sepsis Task Force (comprising 86 pharmacists, nurses, microbiologists, and infection preventionists) for the first national neonatal intensive care unit (NICU)–ASP study across South Africa. The OSU–NCH collaboration expanded the TTT program with partnerships in Lebanon and Latin America.

### Commonwealth Partnerships for Antimicrobial Stewardship (CwPAMS)

Since 2018, through the CwPAMS program, there have been 26 partnerships involving UK institutions and institutions in 8 African countries (Table 1). The partnerships which included >200 multidisciplinary healthcare workers have completed several projects to tackle antimicrobial resistance, mainly through AMS interventions. The CwPAMS has focused on development of tools and facilitation of capacity building for AMS principles, antimicrobial prescribing, and antimicrobial consumption surveillance for 6,831 healthcare workers. These activities have included the generation and strengthening of knowledge and skills, TTT approaches, bidirectional visits, commitment, structures, systems, and leadership among stakeholders in the United Kingdom and Africa. In addition, 29 UK pharmacists who were part of the CwPAMS program took additional leadership training and have graduated as Global Health Pharmacy Fellows.^3^ An equivalent leadership program has been developed for pharmacists based in Africa.

### A UK–India–South Africa social science AMS research study: The ASPIRES study

The multicentric social science Antibiotic use across Surgical Pathways - Investigating, Redesigning and Evaluating Systems (ASPIRES) study, has aimed to optimize antibiotic usage along surgical pathways through research collaboration among the United Kingdom, India, and South Africa.^4^ In one project subteam, structured capacity strengthening was a core value, and onsite training and ongoing mentorship were provided, tailored to the individual and contextual requirements of the recruited pharmacists and nurse early career researchers (ECRs) . In addition, ECRs were trained in AMS, infection prevention and control, as well as specific research methods to evaluate AMS interventions.^4^ Career development was encouraged through creating opportunities for research fellowship and doctoral (PhD) studentships. Initially, training and learning opportunities were mainly focused in a North–South direction, but capacity strengthening resulted in strong and equitable bidirectional collaborations. Cross-country visits provided insight into site-specific cultures and context enabling learning and knowledge exchange. This collaboration forged strong partnerships among the institutions leading to sustainable capacity for AMS-related work and research beyond the original intended course of the project.

### Multicounty virtual collaboration during the COVID-19 pandemic lockdown

At the peak of the COVID-19 pandemic, AMS and infection pharmacists across 9 countries, 5 continents, high-income countries (HICs) and LMICs (Australia, Canada, Lebanon, Nigeria, Qatar, Saudi Arabia, South Africa, United Kingdom, and the United States) collaborated to reinforce the ways in which pharmacists contributed to essential patient care and well-being of the public during the COVID-19 pandemic. They also codelivered a training webinar hosted by the Center for Infectious Disease Research and Policy (CIDRAP) ASP,^5,6^ and they collaborated on a second publication to provide examples of ASP resilience from pharmacists and physicians with different resources and approaches to ASP.

## Our suggestions on how to get started

### Identifying the need and taking the first steps

**Fig. 1.**
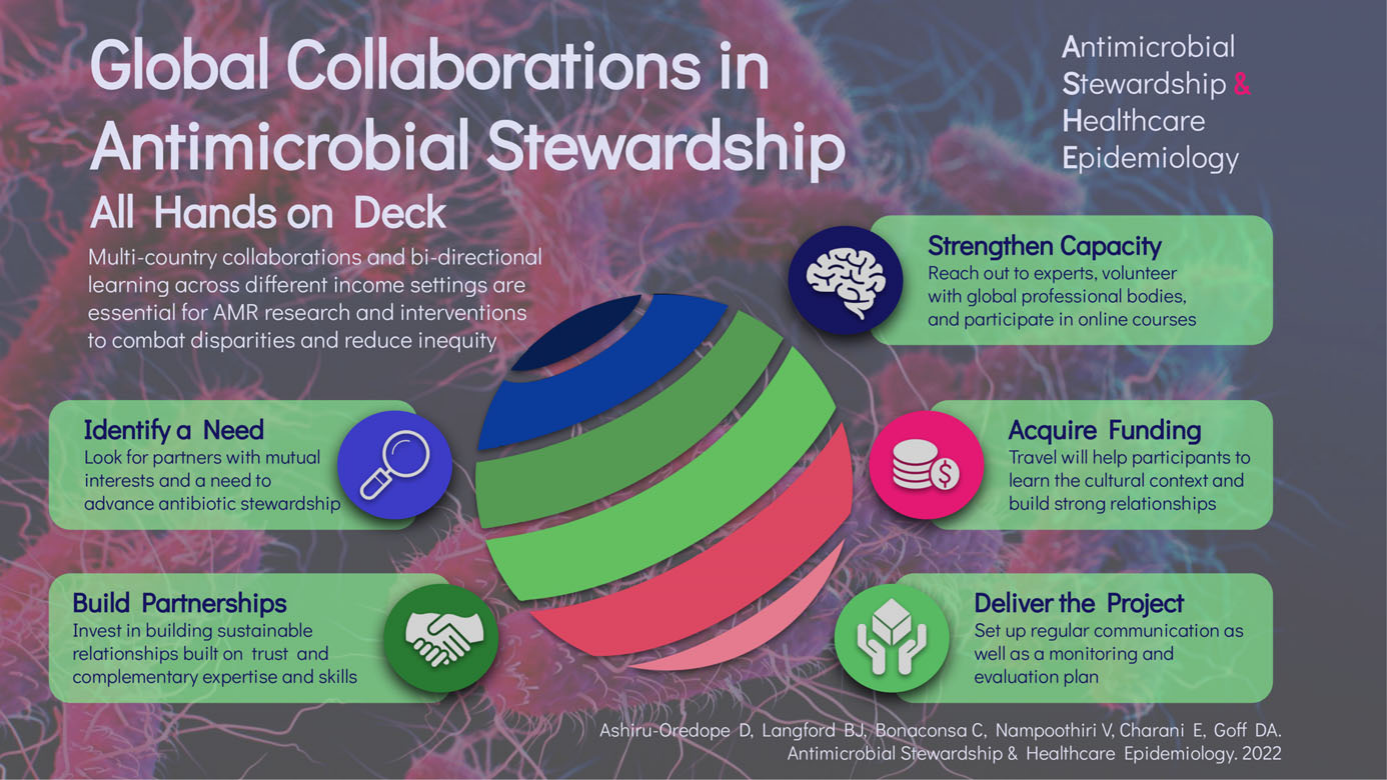
Considerations to advance antimicrobial stewardship through international collaboration.

First, consider some fundamental questions: Will the collaboration result in mutual benefit and how it will progress knowledge and skills? What are the skills, expertise and needs of the teams, organizations, and individuals I intend to work with? How do these match with my own and that of my team, organization, and context? Identifying colleagues who share a mutual interest and the capacity to have the time to work with are important. Once a need and clear gap have been established, as well as a means of how the mutual skills and expertise can address that need, then one can begin to consider the practicalities of collaboration (Fig. 1).

We encourage anyone interested in a global perspective of AMS to take the first step by reaching out to colleagues leading AMS in different settings including universities and professional organizations. AMS research teams within healthcare institutions are usually good places to start. Also, relevant authors of peer review publications or funding bodies can be contacted. One of the simplest ways to start a international AMS collaboration is to connect with people on social media. For example, infectious diseases experts from 4 countries met on Twitter and collaborated to coauthor a paper on the antibiotic topic “Shorter is better and oral is the new IV.” The paper was subsequently posted on Twitter and received 88,900 views in 23 hours.^7^

### Building sustainable relationships and partnerships

In the long run, the health of a collaboration is dependent on the strength of the relationships between the collaborators. Invest in building strong relationships, focusing on mutual trust, interest, and complementary expertise and skills. Ensure that there is adequate commitment from all sides to investing with time and intellectual input into the project. Often, this relationship may benefit from a contractual agreement or memorandum of understanding between partnering organizations. The WHO ESSENCE criteria for capacity strengthening or the Tropical Health and Education Trust (THET) principles for partnership can be used as a framework for establishing equitable partnerships.^8,9^

A recent study using semistructured interviews of global collaborators based in Africa, the Americas, the Eastern Mediterranean, Europe, Southeast Asia, and the Western Pacific highlighted important partnership components.^10^ Key characteristics of successful international partnerships provided by interviewees included personal connections and understanding each other’s programs and systems. These researchers listed open mindedness, adaptability, global citizenship, and cultural and structural awareness as essential qualities for partners and learners.^10^

### Acquiring funding

Although available funding opportunities are highly competitive, applying for them is usually encouraged, especially in the context of international collaborations. Examples of US–UK funding opportunities include Bill and Melinda Gates Foundation, infectious diseases societies, the Centers for Disease Control and Prevention (CDC), pharmaceutical companies, the Wellcome Trust, the UK Aid Fleming Fund, and Health Education England Global Health Fellowships. These websites should be checked regularly for grant funding opportunities, as should other governmental, nongovernmental organizations (NGOs), and foundations. Travel is an important part of global health work, and some humanitarian organizations require experience of working internationally for at least 3 months. Travel is not always required to make important contributions; however, understanding contextual norms in different settings is a valuable skill. Early discussion with partner organizations is ideal to determine what costs need to be covered, how finances will be managed, and whether the involvement of the finance teams of all organizations is needed. The drivers and purposes of collaborations and the beneficiaries must be ascertained. Mutual benefits, including bidirectional learning and capacity strengthening, should be the goals of collaboration. Intentional effort is needed to assure equitable partnerships, especially because funding often flows in one direction from HICs to LMICs.

### Delivering the project

A monitoring and evaluation plan is important to ensure that goals and outcomes are met. Setting up regular communication with all team members, particularly when working remotely or across different sites, is also an important element of a successful collaboration.

### Strengthen global health capacity and skills

Those interested in global collaborations should consider volunteering with global professional bodies such as the International Pharmaceutical Federation (FIP), Doctors of the World, Médecins Sans Frontières, the Red Cross, Save the Children, and many others. Examples of global health fellowship opportunities include the Harkness Fellowships in Health Care Policy and Practice of the Commonwealth Fund and The Churchill Fellowship. Several open university online short courses are available on global health (eg, FutureLearn, OpenLearn, Coursera) and some programs include a global health master’s degree.

As stated by Margaret Mead “Never doubt that a small group of thoughtful, committed citizens can change the world; indeed, it’s the only thing that ever has.” In addition to benefits for patients and the public, there are important personal and professional development benefits for global collaborations to address the threat of AMR. Furthermore, such collaborations broaden the experiences, worldview and leadership skills of antimicrobial stewards who traditionally have not been as involved in global health activities. These efforts will lead to increased ability to implement innovations, will help us learn about health inequalities, and will provide opportunities for bidirectional learning.

## References

[1] Beaglehole R , Bonita R. What is global health? Glob Health Action 2010;3. doi: 10.3402/gha.v3i0.5142. 10.3402/gha.v3i0.5142 PMC2852240 20386617

[2] Goff DA , Bauer KA , Brink A , et al. International train-the-trainer antibiotic stewardship program for pharmacists: implementation, sustainability, and outcomes. J Am Coll Clin Pharm 2020;3:869–876.

[3] Brandish C , Garraghan F , Ng BY , Russell-Hobbs K , Olaoye O , Ashiru-Oredope D , editors. Assessing the impact of a global health fellowship on pharmacists’ leadership skills and consideration of benefits to the National Health Service (NHS) in the United Kingdom. Healthcare (Basel) 2021;9:890. 34356268 10.3390/healthcare9070890 PMC8304379

[4] Veepanattu P , Singh S , Mendelson M , et al. Building resilient and responsive research collaborations to tackle antimicrobial resistance—lessons learnt from India, South Africa, and UK. Int J Infect Dis 2020;100:278–282. 32860949 10.1016/j.ijid.2020.08.057 PMC7449941

[5] Center for Infectious Disease Research & Policy. Global contributions of pharmacists during the COVID-19 pandemic. YouTube website. https://www.youtube.com/watch?v=KQx1-gOptio&t=1s Published online 2021. Accessed December 12, 2022.

[6] Goff DA , Gauthier TP , Langford BJ , et al. Global resilience and new strategies needed for antimicrobial stewardship during the COVID-19 pandemic and beyond. J Am Coll Clin Pharm 2022;5:707–715. 35572210 10.1002/jac5.1622 PMC9087764

[7] Davar K , Clark D , Centor RM , et al. Can the future of ID escape the inertial dogma of its past? The exemplars of shorter is better and oral is the new IV. Open Forum Infect Dis 2022;10:ofac706. 10.1093/ofid/ofac706 PMC9853939 36694838

[8] Special Program for TDR SCI. Planning, monitoring and evaluation framework for research capacity strengthening. World Health Organization website. https://tdr.who.int/publications/m/item/2016-02-15-planning-monitoring-and-evaluation. Published 2016. Accessed March 21, 2023.

[9] Principles of partnership. THET Partnership for Global Health website. https://www.thet.org/principles-of-partnership/. Published online 2019. Accessed December 12, 2022.

[10] Prescott GM , Jonkman L , Crutchley RD , et al. Characteristics of successful international pharmacy partnerships. Pharmacy 2023;11:7. 36649017 10.3390/pharmacy11010007 PMC9844321

